# Supplementary material for: Are carbon emissions trading and green financial instruments synergistic? -Comprehensive quantitative research based on content analysis
Source: PLoS One. 2024 Mar 7;19(3):e0298601. doi: 10.1371/journal.pone.0298601 (PMC10919601; doi:10.1371/journal.pone.0298601)
Supplement: S1 Data — (ZIP) [file pone.0298601.s001.zip › Data/地级市Z_F工作报告.zip!/╔╜╢1⁄2/╟α╡║╩╨/└┤╫╘╣1⁄2╓┌║┼í╛┬φ┐╦╩2╛▌═°í┐_2003.docx]

各位代表:

　　现在,我代表青岛市第十二届人民政府向大会作工作报告,请予审议。并请各位政协委员和其他列席人员提出意见。

　　一、过去五年的回顾

　　过去的五年,是青岛改革开放和现代化建设取得巨大成就的五年。在中共青岛市委的正确领导下,在市人大、政协的监督支持下,本届政府高举邓小平理论伟大旗帜,全面贯彻“三个代表”重要思想,紧紧依靠全市人民,解放思想,实事求是,与时俱进,开拓创新,圆满完成了“九五”计划,顺利实施“十五”计划,市十二届人大一次会议确定的各项任务目标胜利完成,经济综合实力和城市综合竞争力迈上了一个新的台阶。

　　（一）经济社会实现快速发展。经济结构逐步优化,运行质量不断提高。国内生产总值由1997年的797.7亿元增加到去年的1518.2亿元,年均增长14%。三次产业的比重由1997年的14.7∶47.9∶37.4调整到去年的9.6∶50.4∶40。地方财政收入由1997年的44.6亿元增加到去年的100.7亿元,年均增长17.7%。金融机构各项存款和贷款余额由1997年的738.5亿元、650.9亿元增加到去年的1522.6亿元、1304.5亿元。城郊经济快速发展,五个县级市国内生产总值占全市的比重由1997年的35%提高到去年的48%。社会事业繁荣兴旺,科技教育全面发展,公共服务持续改善,社会政治保持稳定,可持续发展能力进一步增强。

　　（二）改革开放取得新的突破。国有企业改革与发展三年目标基本实现,现代企业制度初步确立。所有制结构发生明显变化,各种经济成分共同发展,五年中个体私营经济占全市国内生产总值的比重提高了11.3个百分点。市场体系日趋完善,金融、流通、住房等各项改革不断深化,防范和化解金融风险、整顿规范市场经济秩序的工作成效明显。全方位开放格局基本形成,五年来实际利用外资年均增长21.3%,在我市投资的世界500强企业已有64家;外贸出口年均增长20.2%。累计利用国内资金277亿元。实施“走出去”战略迈出重要步伐。

　　（三）城乡面貌发生显著变化。城市建设五年累计投资377.4亿元,一批基础设施项目建成投产,城市功能逐步完善,城市环境持续改善,空气质量年优良天数达到324天。全市建成区绿化覆盖率由1997年的31.3%提高到38%。人均公共绿地达到10平方米,增加4.6平方米。先后获得“全国创建文明城市工作先进城市”、“国家园林城市”、“中国优秀旅游城市”、“国家环境保护模范城市”、“全国社区建设示范市”等荣誉称号。区域布局不断优化,城乡经济相互促进,小城镇建设步伐加快,城市化水平由1997年的46%提高到54%。

　　（四）人民生活得到明显提高。去年城镇居民人均可支配收入8721元,农民人均纯收入4195元,分别比1997年增长59.3%和61.4%。城乡居民人均储蓄达到10410元,比1997年增加4571元。去年末城镇登记失业率3.8%。国家要求的“两个确保”达到100%。城区和农村居民人均住房使用面积达到17.5平方米和27平方米,分别比1997年增加了4.9平方米和3.3平方米。精神文明建设和民主法制建设成效显著,民主法制意识、城市文明程度和居民素质进一步提高。

　　（五）各项工作迈出新的步伐。政府各部门各单位认真履行职责,勤奋工作,圆满完成了本届政府的各项工作任务。“双拥共建”工作成效显著,各级政府妥善安置转业退伍军人,认真落实拥军优属政策,大力支持部队建设;驻青部队积极支援地方经济建设,承担急难险重任务,参与抢险救灾工作,加强民兵和预备役建设,我市连续四次获得全国“双拥模范城”的光荣称号。中央、省驻青单位全力支持青岛的发展,密切配合青岛的建设,为五年来的变化做出了重要贡献。

　　进入新世纪以来,面对国内外形势发生的新变化、新考验,面对中国加入世界贸易组织带来的新机遇、新挑战,面对改革发展稳定中出现的新情况、新问题,在中共青岛市委的领导下,市政府采取了一系列事关青岛长远发展的重大举措,全市改革开放和经济社会发展呈现出勃勃生机,开创了崭新局面。

　　一是加快构建可持续发展的经济体系。实施了发展港口、海洋、旅游“三大特色经济”的思路。去年港口吞吐量达到1.2亿吨,位居国内港口前五名;集装箱吞吐量达到341万标准箱,位居国内港口前三名。海洋产业快速成长,增加值占全市国内生产总值的12%。旅游经济蓬勃发展,去年接待海内外游客1836.7万人次,旅游总收入150.5亿元。电子家电、石油化工、汽车机车船舶集装箱制造、新材料“四大产业基地”的建设步伐加快。大容量网络冰箱、锂离子电池、CDMA基站等项目相继建成投产,中集集装箱基地开工建设,大炼油项目前期工作有了新突破,新型高分子材料、特种金属材料、海洋生物材料开发取得新进展。现代物流业有了较快发展,一批国际知名的连锁零售企业和物流企业落户青岛。

　　二是加快构筑有特色的现代化国际大城市框架。提出了“高效廉洁的城市管理、可持续发展的经济结构、生态型的城市环境、人才型的居民素质、学习型的城市风气、创新型的城市文化”的城市发展目标,确立了以青岛、黄岛、红岛“三点布局”和以滨海大道为主线“一线展开”的城市发展格局,明确了“经济发展拉动、规划先导、生态标准、突出特色、综合平衡、经营城市”的城市发展原则。青银高速公路青岛段、东西快速路一期、南京路和鞍山路改造、滨海步行道一期等工程相继竣工,流亭机场扩建、同三线青岛段等工程进展顺利。注重运用市场机制配置城市资源,建立了土地储备和公开拍卖招标制度。奥运筹备工作取得了积极进展。

　　三是全面提升城市核心竞争力。实施了政府管理体系“五项工程”,开展了政府部门创建“四型机关”活动,推动了政府职能转变和工作效率的提高。公务员效能投诉电话在改善软环境中发挥积极作用。通过健全法规体系和实行统一执法,不断优化法制环境,促进社会公平、公正和安定。实施了引进人才“111工程”,加快了各类人才的引进。完善了社会保障体系,规范了城乡最低生活保障制度,努力为困难群众排忧解难。大力培植“开放、创新、诚信、和谐、文明、向上”的城市风气,增强了城市的凝聚力和吸引力。

　　四是同步推进改革开放和科技进步。国有企业改革改制后活力明显增强,我市获得中国名牌产品的数量位居全国城市前列。民营经济成为经济增长的新亮点。财税体制改革调动了区市发展财源经济的积极性。外贸出口和招商引资每年迈上一个新台阶。科技创新成效显著,高新技术产业发展和利用高新技术改造传统产业取得新进展。各类园区建设局面喜人,正在成为外商投资的聚集地,成为新技术新产品的孵化区。

　　各位代表!回顾过去的五年,我们欣喜地看到:青岛的经济从未像今天这样繁荣,青岛的城市从未像今天这样美丽,青岛人民的生活从未像今天这样殷实,青岛人民的信心从未像今天这样振奋。取得这样的成就,归功于省委、省政府和中共青岛市委的正确领导,归功于青岛市历届市委、市政府打下的良好基础,归功于市人大、政协、各民主党派与工商联的支持和监督,归功于青岛人民与时俱进、开拓创新的伟大实践,归功于人民解放军驻青部队和武警官兵的无私奉献,归功于中央、省驻青单位的大力支持和密切配合,归功于海内外朋友的关心帮助。在这里,我代表市第十二届人民政府向所有参与、支持、关心青岛发展的同志们、朋友们表示衷心的感谢和崇高的敬意!

　　在充分肯定成就的同时,我们清醒地看到,经济社会发展中还存在着一些问题,政府工作还存在着许多不足。主要是:综合实力还不够强,经济结构还不合理,三产比重仍然偏低,区域发展还不平衡。农业的基础还比较脆弱,农民收入增长比较缓慢。国有企业改革需要深化,部分中小企业生产经营困难,市场竞争力不强。开放水平、投资环境与国内先进城市相比还有差距。城市规划工作亟待加强,建筑精品还不够多,城市管理还不尽如人意,长效管理机制还不完善。就业压力较大,社会保障体系有待健全,一些群众的生活还比较困难,影响社会稳定的因素还比较多。社会事业的发展还不能满足人们的需求,城市文明程度和市民文明素质有待进一步提高。政府职能仍需转变,行政行为仍需规范。个别部门和少数政府工作人员宗旨观念淡漠、服务意识不强、工作作风不正、业务水平不高的问题比较突出,官僚主义、形式主义、以权谋私、与民争利、奢侈浪费等现象依然存在。对此,我们必须高度重视,在今后工作中采取有力措施认真加以解决。

　　二、今后五年的任务目标和今年的工作重点

　　以党的十六大为标志,我国进入了全面建设小康社会、加快推进社会主义现代化的新阶段。青岛作为我国东部沿海开放城市,有邓小平理论和“三个代表”重要思想的指导,有长期发展奠定的良好基础,有中共青岛市委的坚强领导,有各级政府多年积累的丰富经验,有全市人民奋发进取的开拓精神,应该也有条件发展得更快更好,在全面建设小康社会的基础上,率先基本实现现代化,为全国全省做出更大的贡献。

　　实现新世纪新阶段的奋斗目标,今后五年至关重要。我们要进一步增强执政为民的责任感,始终坚持以发展为第一要务,聚精会神搞建设,一心一意谋发展,永不自满,永不懈怠,以良好的精神状态、创新的发展思路、科学的行政方式、务实的工作作风,努力开创青岛改革开放和现代化建设的新局面。

　　今后五年政府工作的主要任务是:坚持以邓小平理论和“三个代表”重要思想为指导,全面贯彻十六大精神,把握“观念更新、机制转换、结构调整”三个关键,突出“外经外贸、高新技术、民营经济”三个亮点,抓好“构建可持续发展的经济体系、构筑有特色的现代化国际大城市框架、提升城市核心竞争力”三件大事,进一步深化各项改革,提高对外开放水平,大力调整经济结构,重视解决“三农”问题,正确处理改革发展稳定的关系,加强社会主义精神文明和民主法制建设,在全面建设小康社会的进程中,加快推进经济发展现代化、城市建设现代化、社会事业现代化、人民生活和人的素质现代化。

　　今后五年经济社会发展的主要预期目标是:

　　———国内生产总值年均增长12%以上,2007年达到3000亿元以上。工业增加值年均增长14%以上,高新技术产品产值年均增长20%以上。第三产业年均增长14.5%。地方财政收入年均增长12.5%。

　　———外贸出口年均增长15%。实际利用外资年均增长20%。全社会固定资产投资年均增长15%。社会消费品零售总额年均增长12%。

　　———港口年吞吐量达到1.7亿吨,集装箱吞吐量达到800万标准箱。旅游总收入年均增长16%。海洋产业增加值占国内生产总值的比重达到20%左右。

　　———城市基础设施和各项功能更加完善。城市化水平达到65%。城区绿化覆盖率达到42%。污水集中处理率达到85%。大气质量保持二级标准,空气质量优良天数逐年增加。

　　———高等教育毛入学率达到30%,高中阶段入学率达到90%,新增劳动力平均受教育年限达到13年。人口自然增长率控制在4‰以内。城市卫生服务体系和农村初级卫生保健体系进一步健全。

　　———城镇居民人均可支配收入、农民人均纯收入年均分别增长7%。城镇人均住房使用面积超过20平方米。社会保障体制更加完善,保障水平不断提高。城镇登记失业率控制在5%左右。

　　我们相信,实现了上述目标,青岛的经济发展将迈上一个新的台阶,城乡面貌将出现一个新的变化,人民生活将提高到一个新的水平。在此基础上,再用十年左右的时间,青岛将建成一个物质文明、政治文明、精神文明协调发展的城市,一个经济更加发展、民主更加健全、科教更加进步、文化更加繁荣、社会更加和谐、人民生活更加殷实的城市。青岛人民将更加信心百倍地迈向实现现代化的宏伟目标。

　　各位代表!实现今后五年的任务目标,首先要扎扎实实地做好第一年的工作,确保开好局、起好步。今年,我们要重点做好以下几个方面的工作:

　　（一）加快结构调整,保持经济快速健康发展

　　坚持以结构调整为主线,以建设国际区域性制造基地为目标,以开放和创新为动力,加快构建可持续发展的经济体系。今年国内生产总值增长13%,地方财政收入增长12.5%。

　　培育三大特色经济。推进“以港兴市”,加快北方国际航运中心建设步伐。青岛港吞吐量完成1.26亿吨,集装箱吞吐量达到400万标准箱。吸引国际著名的港口及船务公司,加快前湾港集装箱码头建设步伐,尽快使前湾港成为国际重要的中转枢纽港。开工建设前湾南港区美国环球货柜集装箱码头项目。前湾北港区集装箱码头合资项目年内签约并正式营运。规划建设液体化工码头及多个业主码头,积极推进油码头三期和煤码头二期改造工程。加快发展航运和仓储、加工、物流等临港产业。年内建成前湾国际物流园,争取开工建设跨国公司临港物流分拨中心。以海洋生物、海洋药物、海洋材料、海水综合利用为重点,培育海洋科技企业,建设海洋产业基地。以“山、海、城、文、商”为主题,深度挖掘和整合旅游资源,高水平编制和实施旅游规划,积极推进旅游大项目建设,加强旅游市场促销,优化旅游发展环境,争创中国最佳旅游城市。

　　建设四大产业基地。大力推进重大项目建设进度,今年要进一步做好大炼油和原油储备项目的前期工作,为尽早开工创造条件。开工建设浦项不锈钢板、青岛电厂二期、集装箱制造及拖车等项目。加快实施海西湾修造船基地、重型卡车和轿车、子午胎等重点技改项目。走新型工业化的路子,以信息化带动工业化,引导企业广泛利用电子信息和网络技术,加强企业研发中心建设,运用高新技术和先进适用技术改造提升传统工业。大力发展品牌经济,实施大集团战略,形成若干以大企业为龙头的产业体系。认真实施《中小企业促进法》,进一步落实相关政策,搞好服务体系建设,引导中小企业参与大企业的资产重组、结构调整和产业配套,加强对外合资合作,提高经营管理水平和技术创新能力。帮助中小企业解决在融资等方面遇到的实际困难,培养一批具有较强市场竞争力的专业化“小巨人”。统筹城乡经济社会发展。更多地关注农村,关心农民,支持农业,把解决“三农”问题放在更加突出的位置来抓。紧紧围绕稳定增加农民收入,进一步调整优化农业和农村经济结构,加快推进农业现代化、农村工业化、农村城镇化、经济国际化进程。大力发展创汇农业、都市农业、观光农业和特色农业,推进农业产业化经营,推广先进适用技术,实施农业科技创新,优化农产品品种品质和优势农产品区域布局,发展农产品精深加工。引导和鼓励城市工业参与农业产业化,扶持重点龙头加工企业,推广“九联模式”,加强农产品质量标准体系和检验检测体系建设,提高农产品质量安全水平。大力发展园区经济,提高乡镇企业的市场竞争能力,搞好重点城镇建设,提高农村城镇化水平,合理引导农业人口向二、三产业转移。完善农民进城就业的服务和管理工作。进一步落实党在农村的基本政策,稳定和完善土地承包关系,按照依法、自愿、有偿的原则进行土地承包经营权流转。完善农村税费改革,巩固农民减负成果。增加对农业的投入,抓好农村基础设施和生态建设,加强耕地林地特别是基本农田保护,搞好水利工程建设,实施大沽河下游整治工程。继续做好扶贫开发工作。

　　大力发展服务业。围绕建设区域性金融中心,培育金融市场,优化金融服务,丰富金融业务,提高融资能力,推进金融安全区建设。继续发展商业零售、餐饮娱乐等传统服务业,积极发展连锁经营、物流配送、电子商务等流通方式以及科技咨询、信息服务、社区服务等新兴产业。编制并实施物流业发展规划,加快国际物流中心建设,建立跨国采购促进中心,吸引跨国公司在青设立商品采购中心和物流配送中心。

　　（二）深化各项改革,为经济发展注入新的活力

　　继续实施国有经济布局和结构调整。深化大中型企业的公司制改革,实现股权多元化,完善法人治理结构。继续推进国有企业重组,更多地吸收民间资本和国外资本,逐步降低国有股的比例。深化企业分配制度改革,完善年薪制试点工作。继续对长期亏损、资不抵债、扭亏无望的企业,实施兼并重组、关闭破产。大力发展民营经济,落实扶持政策,改善发展环境,做好各项服务,支持民营企业的园区建设、结构调整、技术进步和制度创新。

　　按照中央统一部署,深化国有资产管理体制改革,依法设立国有资产管理机构,整合重组国有资产经营公司,积极探索国有资产经营体制和方式。深化投融资体制改革,对市属国有投资公司进行规范改制。全面推进事业单位改革。

　　培育和完善土地、房产、技术、劳动力等各类要素市场,规范和发展产权市场,培育经理人市场和人才市场,发展中介机构和行业协会。通过规范改制、强化培育和政策引导,推动企业上市。深化粮食流通体制改革,培育和规范粮食市场。

　　完善市区财政管理体制,调动各方面建设财源经济的积极性。把对财政的贡献作为对大中型企业经营业绩考核的重要依据,发挥好大中型企业对财源建设的支撑作用。争取更多的大公司总部来青岛注册。加强税收征管,完善征管机制,优化税收服务,促进均衡入库。加强财政监督,重点是非税收入和政府基金的管理。按照建立公共财政框架的要求,调整支出结构,强化支出管理,在市级行政事业单位全面实行部门预算,逐步推行国库集中支付,完善政府采购制度。建立正常的机关事业单位工资增长机制。

　　（三）扩大对外开放,提高经济的国际竞争力

　　继续把对外开放作为经济工作的重中之重,大力推进经济国际化战略,在更大范围、更广领域和更高层次上参与国际经济技术合作和竞争。全年实际利用外资增长35%,外贸出口增长15%以上。

　　创新招商方式,扩大招商领域,调整优化引资结构,全力抓好对韩、日、台等重点国家和重点地区的招商,力争实现新的突破。争取对德国和欧盟招商有新的进展。鼓励民间投资促进机构开展招商活动。鼓励大企业与跨国公司建立战略合作关系,吸引跨国公司来我市设立研发机构、地区总部、制造基地。选择部分优势产业和重点行业,进行整体对外招商。推进服务贸易领域的招商引资,扩大金融、物流、旅游、文化、教育、卫生等领域的对外开放。今年争取引进1—2家外资金融机构。加快开发区、高新区、保税区等重点开放园区的发展,发挥其示范带动作用。争取国家批准设立出口加工区。加强对内招商引资工作,做好对口支援和协作工作。

　　用足用好外经贸政策,在继续扶持大企业的同时,鼓励中小企业和民营企业出口。巩固发展日韩、欧美传统市场,努力开拓俄罗斯、中东、北非、中亚、拉美等新兴市场。大力增加机电产品和高新技术产品出口。提高一般贸易水平,扩大加工贸易规模,争取服务贸易有新突破。吸引国内大企业在青建立出口基地,发展“大进大出”和采购中心式出口。加快实施“走出去”战略,设立专项资金,鼓励企业跨国投资、境外办厂、承包国际工程。

　　继续推进“大通关”工程,建设电子口岸,提高口岸通关速度。探索建立全社会的成本控制体系,降低外商的投资成本。加强外商投资综合服务中心、发展环境投诉中心、公务员效能投诉电话的功能作用,坚持外商投资企业政策法规说明会制度,认真办理外商建议和投诉。

　　（四）以迎办奥运为契机,加快推进城市现代化建设

　　按照经济发展拉动、规划先导、生态标准、突出特色、综合平衡、经营城市的原则,高起点规划,高标准建设,高效能管理,高水平经营,争创“中国人居环境奖”。

　　强化规划龙头作用。组织完成城市发展概念性规划。开展新一轮总体规划修编工作,完成各分区规划和卫星城镇的发展规划,修订城市交通体系规划等专项规划。加快控制性详细规划的编制工作,覆盖率达到30%以上。加强城市风貌保护,延续城市历史文脉。重视城市环境艺术和建筑设计,认真抓好城区干道两侧重点地段的布局规划和单体设计,为城市增添更多的建筑精品。严格实行规划审批行政责任追究制度,完善城市规划法规体系,加强规划的权威性,加大拆违和查处力度。

　　提升城市建设水平。发布实施《青岛奥运行动规划》。完成奥运场馆和浮山湾整体开发项目的规划设计,实施北海船厂搬迁和奥运设施建设,搞好“三湾一线”的保护和整治。完成流亭机场扩建、东西快速路二期、同三线青岛段、前湾港疏港高速公路主体等工程。开工建设滨海大道和东西快速路三期工程。做好海湾大桥前期准备工作,争取早日开工。启动中山路商贸旅游区和东西快速路两侧综合改造,改善城区北部和西部的环境,努力缩小南北差距、协调东西发展。

　　进一步完善“两级政府、三级管理、四级网络”的城市管理体制,健全城市管理法规体系,探讨建立责权利相统一、工作重心下移的长效管理机制。下决心治理局部地区“脏乱差”问题,清除城区卫生死角,改善公用事业和公共设施方面存在的薄弱环节,分区域分路段加强城市的亮化。加快城市管理体制改革,打破垄断,引入竞争,实现城管作业市场化。积极推进城市管理综合执法改革试点。

　　发挥市场机制的作用,注重运用市场手段经营城市。坚持土地利用统一规划、统一征用、统一储备、统一配置、统一管理,强化政府对土地一级市场的高度垄断。加大土地执法力度,扎实开展土地市场清理整顿工作。进一步完善土地储备制度,防止土地供求总量失衡和结构性失衡。探索土地经营的模式和机制,推进土地配置市场化。经营性土地必须实行公开拍卖、招标和挂牌出让。加快市政公用事业产业化步伐,探索基础设施特许经营权拍卖、有偿使用和经营权转让,利用多种方式吸收外资和社会资金用于城市建设。在城建、城管、文化、教育、旅游等领域推出一批经营城市示范项目。

　　（五）实施科教兴市战略,坚持三项基本国策

　　建设国家海洋科学研究中心,抓好国家级试点、示范城市项目和国家重大重点项目,推进重点实验室建设。加强产学研结合,加快重点创新基地建设,把大学科技园、软件园、农业科技示范园、新材料科技园等园区建成技术集成、创新示范的基地,提高关键技术的原创能力。发展科技风险投资公司,促进科技成果产业化。弘扬科学精神,加强科普工作,开展市科技馆项目建设的前期工作。坚持自然科学与社会科学并重,发挥哲学和社会科学研究的积极作用。

　　继续推进素质教育,巩固提高义务教育。普及农村高中阶段教育,全市高中阶段普及率达到85%以上。加大对教育事业的投入,增加对农村教育的支持。鼓励和扶持民办教育,重视发展职业技术教育和职业技能培训。进一步支持驻青高校的发展,加强与国内外高校的合作,引进国内著名高校来我市办学。完善继续教育制度,建立开放性终身教育体系,构建市民终身教育平台。加强教师队伍建设,提高教师的师德和业务水平。

　　实施“人才强市”战略,构筑人才高地。建立人才培养专项资金,创新用人机制,完善人才使用评价体系。重视做好引智工作,培养和引进在国内外有重大影响的学科带头人。继续实施引进人才“111工程”,完善落实人才引进政策,出台人才居住证制度,办好留学人员创业园和博士创业园。

　　深化计划生育综合改革,推进优质服务“三大工程”。完善目标管理责任制,进一步稳定低生育水平,提高人口素质。合理开发和节约使用自然资源,实施中水利用示范工程。树立生态意识,发展循环经济。综合整治水环境、大气环境、重点区域环境。完善城市“绿线”规划编制,实施“绿线”管制制度。规划建设以浮山、北岭山森林绿化为主体的城市山头森林带,以海泊河、张村河、李村河综合治理为主线的河流生态景观带,以滨海岸线绿化保护为核心的滨海特色环境带。合理布局乔木、灌木、草坪的种植。

　　（六）努力增加社会就业,提高群众生活水平

　　进一步完善促进就业的体系和政策,形成劳动者自主择业、市场调节就业、政府促进就业的机制。大力发展非公有制经济和服务业,重视发展劳动密集型产业,努力增加就业机会。引导人们转变就业观念,鼓励和提倡自谋职业、自主创业、灵活就业。以培育“创业小老板”为目标,以自主创业带动就业,年内实现自谋职业7000人。加强对困难群体的就业帮扶,建立再就业援助资金和服务体系。实施过渡性就业工程,新开发交通、城管、社区卫生、治安消防协管员等过渡性岗位6000个。全年实现就业6万人,年末城镇登记失业率控制在4.3%以内。

　　健全和完善社会保障体系。落实“两个确保”和城乡居民最低生活保障制度,完善社会养老保险体系,健全企业补充养老保险制度,推进事业单位养老保险制度改革,探索建立城郊村民社会养老保险制度。加强社会保险扩面征缴工作。完善城镇职工基本医疗保险政策,建立弱势群体社会医疗救助制度,健全医疗保障体系。完善失业保险制度。加大对贫困群体的直接救济与援助力度,健全社会救助体系、灾民救援体系和社会互助体系。做好清理企业欠薪欠费工作。

　　完善相关政策,继续安排好经济适用房建设,年内开工建设经济适用住房200万平方米,竣工60万平方米。运用捆绑开发、土地运作等方式,加大旧城旧村改造力度。

　　（七）加强精神文明建设,推进民主法制建设

　　以思想道德建设为中心环节,以群众喜爱的形式为主要载体,以提高公民素质和城乡文明程度为目的,加强精神文明建设。认真贯彻《公民道德建设实施纲要》,加强社会公德、职业道德、家庭美德教育。大力弘扬蕴涵民族精神、富有时代特征、体现青岛特点的城市精神。以“创服务名牌,树青岛形象”为主题,实施精神文明建设名牌战略,今年要推出3-5个服务品牌。深入开展群众性精神文明创建活动,力争首批进入全国文明城市行列。在全社会进一步强化诚信意识,倡导诚信风尚。深入开展创建学习型城市活动,形成“全民学习、终身学习”的浓厚氛围。

　　全面发展各项社会事业。制定支持公益文化事业、发展文化产业的政策措施。实施精品工程,推出优秀作品。搞好基层文化建设,广泛开展丰富多彩的群众性文化活动。加强文化市场管理,合理开发利用文物资源。做好青岛大剧院前期准备工作,争取尽早开工。搞好农村初级卫生保健工作,推广以大病统筹为主的新型农村合作医疗制度。深化城镇医药卫生体制改革,完善区域卫生规划,优化卫生资源配置,整顿规范公共卫生秩序和医疗服务市场。广泛开展群众性体育运动,提高竞技体育水平,抓好体育设施建设,承办高水平体育赛事,加快体育产业发展。做好民族宗教、外事侨务和对台工作。关心老年人的健康和生活,重视青少年成长成才,推进妇女儿童事业发展,做好慈善事业和残疾人工作。深化国防教育,增强国防观念,加强民兵预备役和人防工作,做好双拥工作,争取第五次获得全国“双拥模范城”荣誉称号。

　　推进以民主选举、民主决策、民主管理、民主监督为主要内容的基层民主政治建设。完善村民自治、城市居民自治以及职工代表大会制度。凡是事关城市发展、涉及群众切身利益的事情,都要让群众知晓、让群众参与、让群众监督。完善社区功能,建设管理有序、文明祥和的新型社区。提高政府依法行政水平。认真执行人民代表大会及其常委会的决议决定,自觉接受法律监督和工作监督。充分重视人民政协、各民主党派及工商联在政治协商、民主监督和参政议政中的作用。做好人大代表和政协委员提出的各项议案、建议、提案办理工作的督查落实。加强政府法制工作,做好行政立法、行政执法监督和行政复议工作。进一步推进行政执法责任制,实施行政执法评议考核制度。拓展法律服务,加强法律援助。深入开展普法教育,提高市民的法律素质,提高政府工作人员的法制素质和依法办事能力。

　　维护社会政治稳定,保障人民群众安全。正确运用政治、经济、行政和法律等手段,妥善处理人民内部矛盾。高度重视做好人民来信来访工作,实行领导负责制,认真解决群众反映的重点热点问题。坚持“打防结合、预防为主”的方针,加强社会治安综合治理,依法严厉打击各种刑事犯罪活动。深入开展与“法轮功”等邪教组织的斗争,坚决扫除各类社会丑恶现象。强化监督检查和安全生产责任制,防止重特大安全生产事故的发生。

　　进一步整顿和规范市场经济秩序。继续抓好“放心工程”,让群众吃得放心、用得放心、住得放心。严厉打击食品、药品、保健品、农业生产资料等领域的制假售假行为。建立规范的市场准入制度和退出机制,健全安全监测网络和长效监督机制,实行严格的全过程质量监控。整顿房产建设、交易、住宅装修和装潢建材市场,提高住宅建设质量,严厉查处侵害消费者权益的不法行为。

　　三、切实加强政府自身建设

　　按照“精神面貌明显变化、服务意识明显增强、工作作风明显改进、工作节奏明显加快、行政效率明显提高”的要求,全面加强政府自身建设,切实转变作风,提高行政效能和服务质量,塑造廉洁、高效、公正的政府形象。

　　深化政府管理体系“五项工程”。切实转变政府职能,着力推进行政体制和管理方式的创新,确保政府工作协调运转、全面提速。公布新一轮行政审批事项清理方案,进一步减少和规范行政审批。提高审批效率,实行审批终身责任追究制度。广泛实行招投标制度。推进政务公开,推广电子政务,完善政府网站、政府公报、新闻发布会等形式。优化决策机制,实行决策公示、听证和专家论证制度。加强审计监督,健全行政效能评估体系。

　　全面建设学习型、创新型、竞争型、服务型的政府机关。政府工作人员特别是领导干部,要坚持用邓小平理论、“三个代表”重要思想武装头脑,学习市场经济、现代科技和法律知识,学习世界贸易组织规则、国际金融、国际化管理服务等方面的知识,不断提高行政能力。创新服务目标、方式和手段,创新考评机制、激励和约束机制。推进行政管理制度改革,依据职责、权力和义务,确定岗位目标责任和考核标准。进一步完善面向社会承诺的服务内容、服务程序、服务标准、承诺时限和违诺责任。规范政府工作人员接待、办事、转接、反馈结果等工作制度。以公众满意度为标准,全面提升服务品质,争创政府服务品牌。切实转变作风,加强廉洁勤政。政府工作人员要按照“八个坚持、八个反对”的要求,务必保持谦虚谨慎、不骄不躁的作风,务必保持艰苦奋斗的作风,坚持执政为民。进一步强化群众观念,增强服务意识,深入基层,深入群众,倾听群众呼声,关心群众疾苦,完善调查研究制度、基层联系点制度、信访接待日制度、与群众对话制度等。倡导求真务实,真抓实干,防止和克服形式主义、官僚主义,各项工作都要抓推进、抓落实、抓见效。认真纠正部门和行业不正之风,切实解决好一些部门、行业和干部中存在的“以权谋私、与民争利、铺张浪费”等问题,解决好农民的不合理负担、中小学乱收费、公路“三乱”、药品生产经营中的不正之风等问题。进一步抓好廉政建设,落实廉政建设责任制,每一个政府工作人员都要严格执行廉洁自律的各项规定,自觉接受人民群众的监督。

　　各位代表!我们已经踏上了全面建设小康社会、加快社会主义现代化建设的新的伟大征程。我们面临着前所未有的机遇和挑战,肩负的任务光荣而艰巨。让我们紧密团结在以胡锦涛同志为总书记的党中央周围,高举邓小平理论伟大旗帜,全面贯彻“三个代表”重要思想,在中共青岛市委的坚强领导下,解放思想,实事求是,与时俱进,开拓创新,为加快青岛的经济社会发展、创造青岛的美好未来而努力奋斗!
